# Annual home‐based HIV testing in the Chókwè Health Demographic Surveillance System, Mozambique, 2014 to 2019: serial population‐based survey evaluation

**DOI:** 10.1002/jia2.25762

**Published:** 2021-07-14

**Authors:** Duncan MacKellar, Ricardo Thompson, Robert Nelson, Isabelle Casavant, Sherri Pals, Juvencio Bonzela, Alicia Jaramillo, Judite Cardoso, Dawud Ujamaa, Stelio Tamele, Victor Chivurre, Inacio Malimane, Ishani Pathmanathan, Kristen Heitzinger, Stanley Wei, Aleny Couto, Alfredo Vergara

**Affiliations:** ^1^ Division of Global HIV and TB National Center for Global Health US Centers for Disease Control and Prevention Atlanta GA USA; ^2^ Chókwè Health Research and Training Center National Institute of Health Chókwè Mozambique; ^3^ US Centers for Disease Control and Prevention Maputo Mozambique; ^4^ Jhpiego at Johns Hopkins University Maputo Mozambique; ^5^ ICF International Atlanta GA USA; ^6^ District Directorate of Public Health Chókwè Mozambique; ^7^ Provincial Directorate of Public Health Xai‐Xai Mozambique; ^8^ Mozambique Ministry of Health Maputo Mozambique

**Keywords:** home‐based HIV testing and counselling, HIV diagnostic coverage, prevalence of undiagnosed HIV infection, Mozambique

## Abstract

**Introduction:**

WHO recommends implementing a mix of community and facility testing strategies to diagnose 95% of persons living with HIV (PLHIV). In Mozambique, a country with an estimated 506,000 undiagnosed PLHIV, use of home‐based HIV testing services (HBHTS) to help achieve the 95% target has not been evaluated.

**Methods:**

HBHTS was provided at 20,000 households in the Chókwè Health Demographic Surveillance System (CHDSS), Mozambique, in annual rounds (R) during 2014 to 2019. Trends in prevalence of HIV infection, prior HIV diagnosis among PLHIV (diagnostic coverage), and undiagnosed HIV infection were assessed with three population‐based surveys conducted in R1 (04/2014 to 04/2015), R3 (03/2016 to 12/2016), and R5 (04/2018 to 03/2019) of residents aged 15 to 59 years. Counts of patients aged ≥15 years tested for HIV in CHDSS healthcare facilities were obtained from routine reports.

**Results:**

During 2014 to 2019, counsellors conducted 92,512 home‐based HIV tests and newly diagnosed 3711 residents aged 15 to 59 years. Prevalence of HIV infection was stable (R1, 25.1%; R3 23.6%; R5 22.9%; *p*‐value, 0.19). After the first two rounds (44,825 home‐based tests; 31,717 facility‐based tests), diagnostic coverage increased from 73.8% (95% CI 70.3 to 77.2) in R1 to 93.0% (95% CI 91.3 to 94.7) in R3, and prevalence of undiagnosed HIV infection decreased from 6.6% (95% CI 5.6 to 7.5) in R1 to 1.7% (95% CI 1.2 to 2.1) in R3. After two more rounds (32,226 home‐based tests; 46,003 facility‐based tests), diagnostic coverage was 95.4% (95% CI 93.7 to 97.1) and prevalence of undiagnosed HIV infection was 1.1% (95% CI 0.7 to 1.5) in R5. Prevalence of having last tested at home was 12.7% (95% CI 11.3 to 14.0) in R1, 45.2% (95% CI 43.4 to 47.0) in R3, and 41.4% (95% CI 39.5 to 43.2) in R5, and prevalence of having last tested at a healthcare facility was 45.3% (95% CI 43.3 to 47.3) in R1, 40.1% (95% CI 38.4 to 41.8) in R3, and 45.2% (95% CI 43.3 to 47.0) in R5.

**Conclusions:**

HBHTS successfully augmented facility‐based testing to achieve HIV diagnostic coverage in a high‐burden community of Mozambique. HBHTS should be considered in sub‐Saharan Africa communities striving to diagnose 95% of persons living with HIV.

## 1 Introduction

To achieve HIV epidemic control, countries are striving to diagnose 95% of persons living with HIV (PLHIV), initiate and retain on antiretroviral therapy (ART) 95% of those diagnosed, and ensure 95% of those on ART are virally suppressed (95‐95‐95) [1]. Of the 95‐95‐95 targets, diagnosing 95% of PLHIV has for many countries been the most challenging to reach [2, 3, 4]. In Mozambique, an estimated 77% of 2.2 million PLHIV of all ages had received an HIV diagnosis by the end of 2019, including 86% of women and 66% of men aged ≥15 years [4]. In 2020, estimated diagnostic coverage in Mozambique was <90% in nine of 11 provinces, and was particularly low among women and men aged 15 to 24 years nationally (women, 71%; men 45%) [5, 6].

To achieve 95% diagnostic coverage, WHO recommends implementing a mix of testing strategies including HBHTS in high‐burden communities [7]. Systematic reviews of over 30 studies suggest that HBHTS is cost‐effective and superior to other strategies in testing a large majority of residents in geographical populations (testing coverage) and in diagnosing PLHIV sooner after acquiring HIV [8, 9, 10, 11]. Few studies, however, have evaluated if HBHTS, in addition to standard testing in healthcare facilities, can achieve 95% diagnostic coverage; none have been conducted in Mozambique [12, 13].

To assess the potential of HBHTS to augment facility‐based testing to achieve 95% diagnostic coverage and reduce the prevalence of undiagnosed HIV infection in a high‐burdened population, annual HBHTS was offered at all households in the Chókwè Health Demographic Surveillance System (CHDSS) during 2014 to 2019. Located in Gaza Province of southern Mozambique, CHDSS conducts annual demographic and vital‐events surveillance of approximately 100,000 residents in Chókwè town and seven rural villages, representing approximately half of the population in Chókwè District.

In this paper, we report trends in the annual number of residents aged 15 to 59 years who were tested at home and received an HIV diagnosis for the first time (new diagnosis), diagnostic coverage among PLHIV, and population prevalence of undiagnosed HIV infection, by age group, sex, and HIV‐risk characteristics. Evaluating the distribution and trends of undiagnosed HIV infection is important to identify PLHIV populations underserved by HBHTS and standard facility‐based testing strategies. Our findings may be helpful to countries considering the targeted application of HBHTS to reduce disparities in undiagnosed HIV infection and to help diagnose 95% of PLHIV in high‐burden communities.

## 2 Methods

### 2.1 HBHTS intervention

After a partial rollout during 2013, comprehensive door‐to‐door HBHTS was conducted in five annual rounds (R1 to R5) from January 2014 through March 2019. During each round, lay counsellors certified to provide rapid HIV testing and counselling visited approximately 20,000 homes that compose the CHDSS and offered HBHTS to all encountered household members. Rapid HIV testing was conducted in accordance with national guidelines: the screening test was Determine, and the second test, Uni‐Gold, was conducted if Determine was reactive [14]. Participants with reactive results for both tests were considered HIV positive [14]. National testing guidelines did not change during R1 to R5. All HBHTS clients were provided condoms, risk‐reduction counselling, and referral for medical care if needed. HIV‐positive clients were referred for immediate HIV care and re‐visited up to five times over six months to provide supportive counselling for early ART initiation and retention.

### 2.2 Facility‐based testing

During R1 to R5, facility‐based rapid HIV testing in CHDSS was implemented in accordance with national strategic plans supported by the United States President’s Emergency Plan for AIDS Relief [15]. HIV testing at healthcare facilities was conducted in accordance with national guidelines [14]. The number of HIV tests conducted of patients aged ≥15 years at all nine healthcare facilities located in CHDSS during R1 to R5 was obtained from routine Ministry of Health reports. New HIV diagnoses among CHDSS residents tested at healthcare facilities were not measured and are not reportable.

### 2.3 HBHTS evaluation

We conducted three population‐based, cross‐sectional surveys in rounds 1, 3, and 5 to evaluate trends in the prevalence of HIV risk and testing behaviour, prior HIV diagnosis among PLHIV, and undiagnosed HIV infection among CHDSS residents. For each survey round, a separate random sample of households was drawn from the most recent census of CHDSS households in Chókwè town (urban) and seven rural villages (Appendix S1). All households in the updated census were eligible for random selection, including those selected for surveys in prior rounds. The number of households selected for each survey was expected to yield a target sample of 4760 participants that would provide >90% power to detect expected increases in diagnostic coverage among HIV‐positive residents, by urban or rural residence. Because of lower than expected participation rates, attributed in part to some household members working in South Africa, the number of randomly selected households was increased from 2805 in R1 to 4613 and 4577 in R3 and R5, respectively [16]. All contacted members aged 15 to 59 years of selected households were invited to participate in an interview. Interviews were conducted in Shangaan or Portuguese and included standard measures on demographics, HIV risk behaviours, and location and results of their last HIV test. After the interview, consenting participants were tested for HIV in accordance with national guidelines [14]. For participants who tested HIV positive, 1 mL of whole blood was collected and processed into dried blood spots (DBS). Viral load testing was performed on DBS with the Roche COBAS® AmpliPrep/COBAS® TaqMan® HIV‐1 Test at the US Centers for Disease Control and Prevention (CDC) (R1 to R3) and the Instituto Nacional de Saúde, Marracuene, Mozambique (R4 to R5) [17, 18].

### 2.4 Outcome definitions

Prior HIV diagnosis among HIV‐positive survey participants was defined as either (1) reporting having tested HIV positive previously, (2) having tested HIV positive at home in a prior round, or (3) testing HIV positive and having a suppressed viral load (HIV‐1 RNA concentration <1000 copies/μL). Undiagnosed HIV infection (new HIV diagnosis) among HIV‐positive participants was defined as not having received a prior diagnosis.

### 2.5 Statistical analysis

For each round, we report the number of home‐based tests conducted and the percentage of new HIV diagnoses, by sex, age group, and urban or rural residence. In the two rounds before R3 and R5 surveys, we also report the number of tests conducted and new diagnoses (HBHTS only) at CHDSS homes and healthcare facilities. For each of the three cross‐sectional surveys, census‐weighted prevalence outcomes and 95% confidence intervals (CI) were calculated adjusting for household clustering. Differences in prevalence of risk behaviour, prior HIV testing, and HIV infection were evaluated using Rao‐Scott Chi‐square tests. Demographic, prior‐testing, and risk‐behaviour disparities in undiagnosed HIV infection were evaluated comparing R3 with R1, and R5 with R3. Prevalence ratios (PR) and adjusted prevalence ratios (APR) were estimated using generalized estimating equation models with a log link and empirical sandwich standard errors to account for correlation within households and repeat participation of household members across survey rounds. For all surveys, we divided participants and census data into cells defined by age group, sex, and urban or rural residence, and computed weights by dividing the percentage of census participants in each cell by the percentage of survey participants. We incorporated weights into all statistical tests and models to address under coverage of the sample and survey nonresponse by age, sex, and residence. All analyses were conducted using SAS (version 9.4, SAS Institute).

## 3 Results

### 3.1 HBHTS intervention

**Figure 1:**
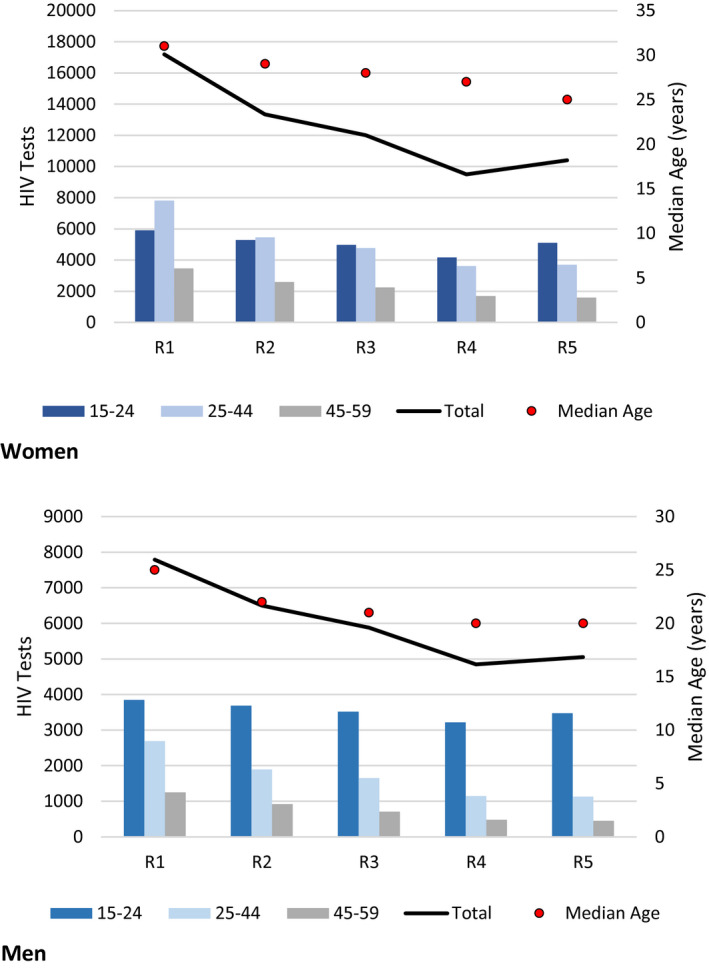
Home‐based HIV tests conducted among women and men, by age group, median age of residents tested, and round (R), Chókwè Health Demographic Surveillance System, Chókwè Mozambique, 2014 to 2019. R1 = 04/2014 to 04/2015; R2 = 05/2015 to 01/2016; R3 = 03/2016 to 12/2016; R4 = 03/2017 to 11/2017; R5 = 04/2018 to 03/2019.

**Figure 2:**
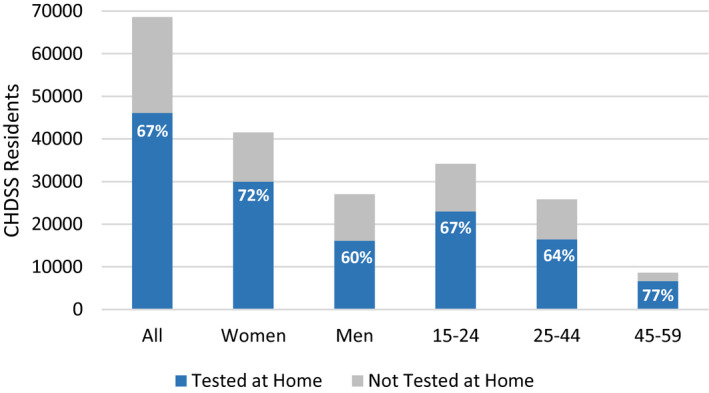
Proportion of residents aged 15 to 59 years who tested at home at least once, by sex and age group in years, Chókwè Health Demographic Surveillance System, Chókwè District, Mozambique, 2014 to 2019.

During R1 to R5, counsellors conducted 92,512 home‐based HIV tests, including 30,072 (32.5%) among men and 43,214 (46.7%) among residents aged 15 to 24 years. Total (monthly) tests declined from 24,979 (1921) in R1 to 15,461 (1288) in R5 (Table S1, Figure 1). Trends in tests per round were similar by sex and age group (Figure 1). From R1 to R5, the median age among residents tested at home decreased from 31 (interquartile range (IQR) 21 to 41) to 25 (IQR 19 to 38) years among women, and from 25 (IQR 18 to 38) to 20 (IQR 17 to 28) years among men. Of 68,620 persons aged 15 to 59 years who ever resided in CHDSS, 46,090 (67.2%) had tested at home at least once (Figure 2).

**Figure 3:**
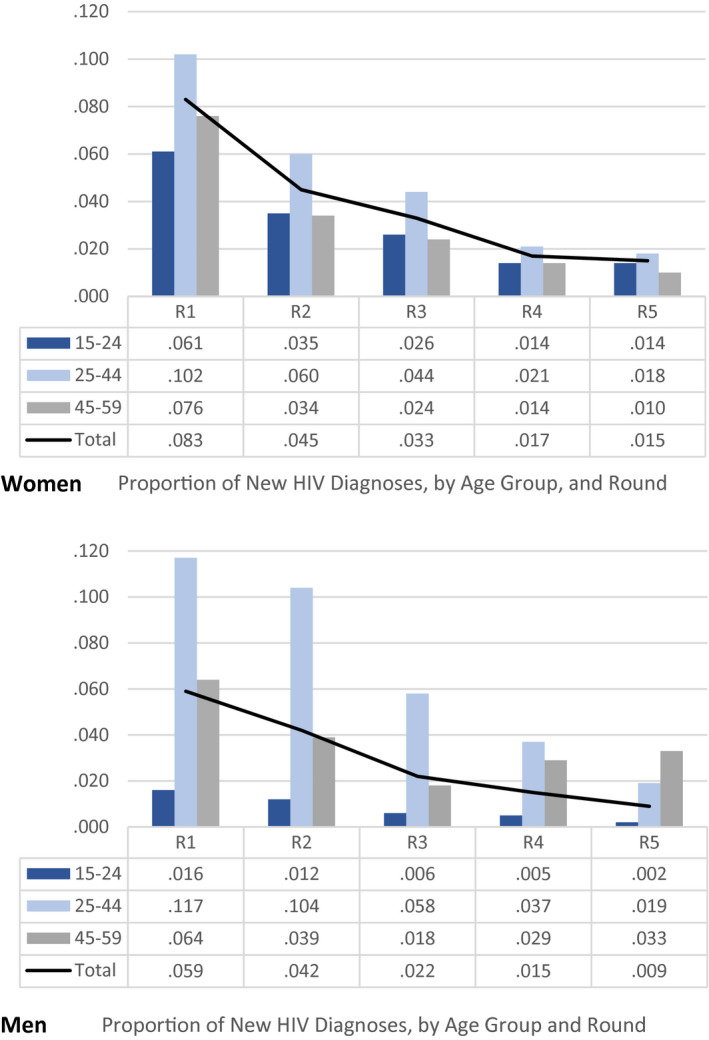
Proportion of new HIV diagnoses among women and men tested for HIV at home, by age group and round (R), Chókwè Health Demographic Surveillance System, Chókwè District, Mozambique, 2014 to 2019. R1 = 04/2014 to 04/2015; R2 = 05/2015 to 01/2016; R3 = 03/2016 to 12/2016; R4 = 03/2017 to 11/2017; R5 = 04/2018 to 03/2019.

During R1 to R5, 3711 residents tested at home received a new HIV diagnosis, including 980 men and 964 persons aged 15 to 24 years. The percentage of new diagnoses of tests conducted declined from 7.5% (R1) to 1.3% (R5) overall, with similar trends for women and men (Figure 3). Among residents aged 15 to 24 years, the percentage of new diagnoses in each round was three to seven times higher among women (6.1% to 1.4%) than men (1.6% to 0.2%). Among residents aged ≥25 years, the proportion of new diagnoses in each round was similar among women and men (Figure 3).

During R1 to R2, 59.1% (32,682/55,282) of all residents had tested at home at least once, 48% (44,825/92,512) of all home‐based tests had been conducted, and 74% (2755/3711) of all residents newly diagnosed at home had been identified and referred to HIV care. During R3 to R4, 32,226 home‐based tests had been conducted and an additional 20% (755/3711) of all residents newly diagnosed at home had been identified and referred to HIV care (Table S1).

### 3.2 Facility‐based testing

During R1 to R5, of 113,327 HIV tests among patients aged ≥15 years at CHDSS healthcare facilities, 31,717 (28%) were conducted during R1 to R2 and 46,003 (41%) were conducted during R3 to R4 (Table S2).

### 3.3 HBHTS evaluation

**Figure 4:**
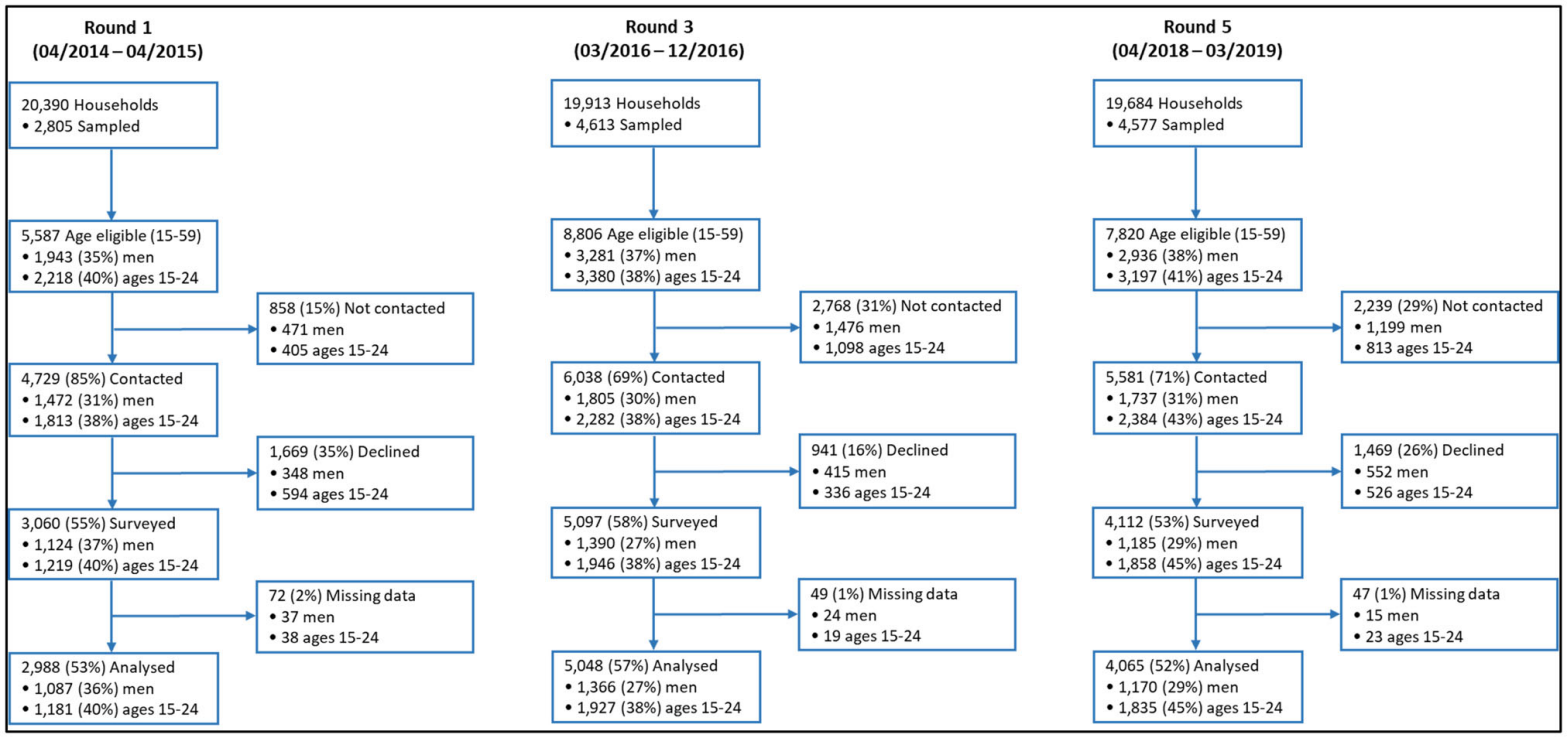
Participation in cross‐sectional household surveys, Chókwè Health Demographic Surveillance System, Chókwè District, Mozambique, 2014 to 2019.

Of residents aged 15 to 59 years in sampled households, 2988, 5048, and 4065 were interviewed and contributed complete analysis records in R1, R3, and R5, respectively (Figure 4). In R3 and R5, men aged 15 to 59 years represented 37% and 38% of members of sampled households, and 27% and 29% of analysed records, respectively. From R1 to R5, prevalence of having ≥1 sexual partners, sometimes or never using condoms, and weekly or daily alcohol use decreased, and prevalence of having tested for HIV in the past year increased (Table 1). Prevalence of having last tested for HIV at a healthcare facility was stable across rounds (45.3% to 45.2%), whereas the prevalence of having last tested at home increased from 12.7% in R1, to 45.2% and 41.4% in R3 and R5, respectively (Table 1).

**Table 1:** Prevalence of HIV risk and testing behaviour among residents aged 15 to 59 years, Chókwè Health Demographic Surveillance System, Chókwè District, Mozambique, 2014 to 2019

	Round 1 (04/2014 to 04/2015)		Round 3 (03/2016 to 12/2016)		Round 5 (04/2018 to 03/2019)		*p*‐value^b^
	n	% (95% CI)^a^	n	% (95% CI)^a^	n	% (95% CI)^a^	
Total	2988	–	5048	–	4065	–	–
Sex							–
Women	1901	62.1 (60.2 to 63.9)	3682	62.1 (60.4 to 63.7)	2895	61.7 (60.0 to 63.4)	
Men	1087	37.9 (36.1 to 39.8)	1366	37.9 (36.3 to 39.6)	1170	38.3 (36.6 to 40.0)	
Age group (years)							–
15 to 24	1181	41.1 (39.2 to 42.9)	1927	41.1 (39.5 to 42.6)	1835	40.9 (39.2 to 42.6)	
25 to 44	1259	42.5 (40.5 to 44.5)	2091	42.5 (40.8 to 44.2)	1500	42.6 (40.7 to 44.6)	
45 to 59	548	16.4 (15.1 to 17.7)	1030	16.5 (15.3 to 17.6)	730	16.5 (15.2 to 17.8)	
Residence							–
Chókwè town	1387	63.1 (60.6 to 65.5)	1840	62.8 (60.7 to 64.8)	1587	62.9 (60.7 to 65.2)	
District villages	1601	36.9 (34.5 to 39.4)	3208	37.2 (35.2 to 39.3)	2478	37.1 (34.8 to 39.3)	
Sexual partners in past 12 months							<0.0001
0	461	15.1 (13.7 to 16.5)	1040	19.4 (18.1 to 20.7)	1028	23.1 (21.6 to 24.5)	
1	1751	58.0 (56.2 to 59.9)	3195	59.8 (58.1 to 61.5)	2806	69.7 (68.1 to 71.4)	
>1	773	26.9 (25.1 to 28.7)	801	20.8 (19.2 to 22.3)	229	7.2 (6.1 to 8.3)	
Condom use in past 12 months							<0.0001
No sexual partners/always	616	21.5 (19.8 to 23.1)	1055	23.7 (22.1 to 25.2)	1073	27.0 (25.3 to 28.6)	
Sometimes/never	2368	78.5 (76.9 to 80.2)	3925	76.3 (74.8 to 77.9)	2970	73.0 (71.4 to 74.7)	
Alcohol use in past 3 months							<0.0001
Never/monthly	2575	85.7 (84.3 to 87.2)	4488	87.7 (86.4 to 88.9)	3788	91.7 (90.6 to 92.9)	
Daily/weekly	398	14.3 (12.8 to 15.7)	517	12.3 (11.1 to 13.6)	262	8.3 (7.1 to 9.4)	
Prior HIV test							<0.0001
<1 year	1104	39.1 (37.1 to 41.0)	1972	42.5 (40.7 to 44.2)	2379	60.0 (58.0 to 61.9)	
1 to 4 years	911	30.7 (28.9 to 32.5)	2484	47.1 (45.3 to 48.9)	1174	28.8 (27.0 to 30.6)	
≥5 years/no prior HIV test	896	30.3 (28.5 to 32.1)	535	10.4 (9.4 to 11.4)	470	11.2 (10.1 to 12.4)	
Location of last HIV test							<0.0001
Never tested	697	22.6 (21.0 to 24.3)	333	7.3 (6.4 to 8.1)	241	5.6 (4.8 to 6.3)	
District hospital/clinic	1347	45.3 (43.3 to 47.3)	2023	40.1 (38.4 to 41.8)	1849	45.2 (43.3 to 47.0)	
District home	386	12.7 (11.3 to 14.0)	2317	45.2 (43.4 to 47.0)	1604	41.4 (39.5 to 43.2)	
Other location	535	19.4 (17.8 to 21.0)	336	7.5 (6.5 to 8.5)	350	7.9 (6.9 to 8.9)	
HIV positive							0.1901
No	2230	74.9 (73.1 to 76.7)	3717	76.4 (75.0 to 77.9)	3119	77.1 (75.4 to 78.7)	
Yes	758	25.1 (23.3 to 26.9)	1331	23.6 (22.1 to 25.0)	946	22.9 (21.3 to 24.6)	

CI, confidence interval.

^a^ All estimates were weighted to the CHDSS census population by age group, sex, and urban (Chókwè town) or rural (district villages) residence, and adjusted for within‐household correlation

^b^ Rao‐Scott chi‐square test of differences in prevalence estimates across survey rounds, not reported for variables used for census‐based weights.

The prevalence of HIV infection (25.1% to 22.9%) was stable across rounds, and consistently higher among women than men, and residents aged 35 to 44 years than all other age groups (Tables 1 and 2). Among residents aged 15 to 24 years, HIV prevalence across survey rounds was at least three‐fold higher among women (11.9% to 8.3%) than men (2.7% to 2.2%) (Table 2). The prevalence of prior HIV diagnosis among PLHIV increased from 73.8% in R1, to 93.0% and 95.4% in R3 and R5, respectively. In R5, prevalence of prior HIV diagnosis was >90% in all subgroups except those aged 15 to 24 years (88.5%) (Table 2).

**Table 2:** Prevalence of HIV infection and diagnosed HIV infection among HIV‐positive residents aged 15 to 59 years, Chókwè Health Demographic Surveillance System, Chókwè District, Mozambique, 2014 to 2019

	Round 1 (04/2014 to 04/2015)				Round 3 (03/2016 to 12/2016)				Round 5 (04/2018 to 03/2019)			
	Participants	HIV positive	HIV prevalence	Prior HIV diagnosis^a^	Participants	HIV positive	HIV prevalence	Prior HIV Diagnosis^a^	Participants	HIV positive	HIV prevalence	Prior HIV diagnosis^a^
	n	n	% (95% CI)	% (95% CI)	n	n	% (95% CI)	% (95% CI)	n	n	% (95% CI)	% (95% CI)
Total	2988	758	25.1 (23.3 to 26.9)	73.8 (70.3 to 77.2)	5048	1331	23.6 (22.1 to 25.0)	93.0 (91.3 to 94.7)	4065	946	22.9 (21.3 to 24.6)	95.4 (93.7 to 97.1)
Sex											
Women	1901	558	28.8 (26.6 to 30.9)	78.2 (74.6 to 81.8)	3682	1108	28.1 (26.5 to 29.8)	93.6 (91.9 to 95.3)	2895	796	27.3 (25.4 to 29.2)	95.8 (94.0 to 97.6)
Men	1087	200	19.1 (16.4 to 21.7)	62.8 (55.3 to 70.2)	1366	223	16.2 (13.8 to 18.5)	91.2 (87.0 to 95.5)	1170	150	15.9 (13.3 to 18.4)	94.1 (89.9 to 98.3)
Age group (years)											
15 to 24	1181	89	7.8 (6.2 to 9.4)	51.6 (41.0 to 62.3)	1927	155	7.6 (6.3 to 8.8)	84.3 (78.3 to 90.3)	1835	108	5.5 (4.4 to 6.7)	88.5 (81.3 to 95.7)
25 to 34	741	253	33.0 (29.4 to 36.6)	72.0 (66.0 to 78.0)	1084	361	28.7 (25.5 to 31.8)	88.2 (84.1 to 92.4)	788	266	30.0 (26.3 to 33.6)	95.3 (92.2 to 98.4)
35 to 44	518	244	46.2 (41.4 to 50.9)	79.4 (74.1 to 84.8)	1007	453	42.4 (38.3 to 46.4)	97.6 (96.2 to 99.1)	712	302	39.5 (35.1 to 44.0)	96.5 (93.8 to 99.3)
45 to 59	548	172	33.2 (28.7 to 37.8)	80.6 (73.7 to 87.4)	1030	362	35.1 (31.3 to 39.0)	97.1 (94.8 to 99.4)	730	270	37.1 (32.7 to 41.6)	96.6 (93.9 to 99.3)
Sex and age group											
Women, 15 to 24	641	77	11.9 (9.3 to 14.5)	55.0 (43.7 to 66.3)	1171	136	11.8 (9.8 to 13.8)	82.8 (76.1 to 89.6)	1103	92	8.3 (6.5 to 10.0)	85.9 (77.3 to 94.6)
Women, 25 to 44	888	363	41.1 (37.7 to 44.6)	81.5 (77.4 to 85.6)	1697	686	38.7 (36.0 to 41.4)	94.8 (92.8 to 96.8)	1201	484	38.3 (35.2 to 41.4)	96.6 (94.6 to 98.6)
Women, 45 to 59	372	118	32.1 (27.0 to 37.2)	85.5 (78.7 to 92.3)	814	286	34.6 (30.8 to 38.4)	97.6 (95.6 to 99.7)	591	220	38.0 (33.5 to 42.4)	98.1 (96.3 to 100)
Men, 15 to 24	540	12	2.7 (1.2 to 4.3)	–	756	19	2.4 (1.2 to 3.6)	–	732	16	2.2 (1.0 to 3.4)	–
Men, 25 to 44	371	134	33.9 (28.8 to 38.9)	62.8 (54.0 to 71.6)	394	128	26.5 (21.6 to 31.4)	88.7 (82.6 to 94.7)	299	84	26.0 (20.8 to 31.3)	94.0 (88.5 to 99.5)
Men, 45 to 59	176	54	35.7 (27.0 to 44.5)	70.9 (56.2 to 85.5)	216	76	36.2 (28.2 to 44.3)	96.0 (90.6 to 100)	139	50	35.4 (26.2 to 44.5)	93.1 (85.3 to 100)
Residence											
Chókwè town	1387	343	24.9 (22.5 to 27.4)	74.5 (69.8 to 79.3)	1840	424	22.3 (20.2 to 24.4)	93.7 (91.3 to 96.1)	1587	330	21.8 (19.5 to 24.2)	95.1 (92.5 to 97.7)
District villages	1601	415	25.4 (23.1 to 27.7)	72.5 (67.9 to 77.0)	3208	907	25.7 (24.1 to 27.4)	91.9 (89.6 to 94.3)	2478	616	24.8 (22.9 to 26.7)	95.7 (93.9 to 97.5)

CI, confidence interval.

^a^ Of HIV‐positive participants. Prior HIV diagnosis = (1) reporting having tested HIV positive previously to standard interview questions, (2) having tested HIV positive at home in a prior round, or (3) having an HIV‐1 RNA concentration <1000 copies/μL. All estimates were weighted to the CHDSS census population by age group, sex, and urban (Chókwè town) or rural (CHDSS villages) residence, and adjusted for within‐household correlation. Reported for sex and age groups with at least 50 HIV‐positive participants.

Compared with R1, the prevalence of undiagnosed HIV infection in R3 declined 75% overall (Prevalence Ratio (PR) 0.25; 95% CI 0.19 to 0.34), and by at least 66% (PR ≤ 0.34) among all sex, age, residence, and risk‐behaviour subgroups (Table 3). Compared with R3, prevalence of undiagnosed infection in R5 was not statistically significantly lower overall and for all subgroups except residents in rural villages and those who reported not testing in the past five years (Table 3).

**Table 3:** Prevalence of undiagnosed HIV infection among residents aged 15 to 59 years, and intra‐ and inter‐survey prevalence ratios, Chókwè Health Demographic Surveillance System, Chókwè District, Mozambique, 2014 to 2019

	Round 1 (04/2014 to 04/2015)			Round 3 (03/2016 to 12/2016)			Round 5 (04/2018 to 03/2019)			Round 3 vs. Round 1	Round 5 vs. Round 3
	**n** ^a^	% (95% CI)^b^	APR (95% CI)^c^	n	% (95% CI)^b^	APR (95% CI)^c^	n	% (95% CI)^b^	APR (95% CI)^c^	PR (95% CI)^c^	PR (95% CI)^c^
Total	2983	6.6 (5.6 to 7.5)	–	5048	1.7 (1.2 to 2.1)	–	4065	1.1 (0.7 to 1.5)	–	0.25 (0.19 to 0.34)	0.64 (0.41 to 1.01)
Sex											
Women	1896	6.2 (5.1 to 7.3)	Referent	3682	1.8 (1.3 to 2.3)	Referent	2895	1.1 (0.6 to 1.7)	Referent	0.29 (0.21 to 0.40)	0.63 (0.38 to 1.07)
Men	1087	7.1 (5.3 to 8.9)	1.3 (0.9 to 1.7)	1366	1.4 (0.7 to 2.1)	0.8 (0.5 to 1.6)	1170	0.9 (0.3 to 1.6)	0.9 (0.4 to 2.2)	0.20 (0.11 to 0.35)	0.66 (0.27 to 1.59)
Age group (years)											
15 to 24	1181	3.8 (2.6 to 4.9)	Referent	1927	1.2 (0.7 to 1.7)	Referent	1835	0.6 (0.2 to 1.1)	Referent	0.32 (0.19 to 0.53)	0.54 (0.24 to 1.18)
25 to 44	1255	9.3 (7.6 to 11.0)	2.5 (1.8 to 3.6)	2091	2.4 (1.6 to 3.1)	2.0 (1.1 to 3.4)	1500	1.4 (0.7 to 2.1)	2.2 (0.9 to 4.9)	0.25 (0.17 to 0.37)	0.59 (0.32 to 1.09)
45 to 59	547	6.4 (4.0 to 8.9)	1.8 (1.1 to 2.9)	1030	1.0 (0.2 to 1.8)	0.8 (0.3 to 2.1)	730	1.3 (0.3 to 2.3)	1.9 (0.6 to 5.9)	0.16 (0.07 to 0.38)	1.23 (0.40 to 3.76)
Residence											
Chókwè town	1385	6.3 (5.0 to 7.7)	Referent	1840	1.4 (0.9 to 2.0)	Referent	1587	1.1 (0.5 to 1.7)	Referent	0.22 (0.14 to 0.35)	0.75 (0.38 to 1.48)
District villages	1598	7.0 (5.6 to 8.3)	1.1 (0.9 to 1.5)	3208	2.1 (1.5 to 2.7)	1.5 (0.9 to 2.5)	2478	1.1 (0.6 to 1.5)	1.0 (0.5 to 2.0)	0.30 (0.21 to 0.43)	0.51 (0.31 to 0.86)
Sexual partners in past 12 months											
0	461	4.2 (2.2 to 6.2)	Referent	1040	0.5 (0.1 to 0.9)	Referent	1028	0.5 (0.0 to 1.0)	Referent	0.12 (0.05 to 0.32)	0.90 (0.24 to 3.34)
1	1747	6.7 (5.5 to 7.9)	1.2 (0.7 to 2.1)	3195	1.9 (1.3 to 2.4)	2.8 (1.1 to 7.0)	2806	1.3 (0.7 to 1.8)	2.3 (0.7 to 7.2)	0.28 (0.20 to 0.39)	0.68 (0.41 to 1.14)
>1	772	7.6 (5.5 to 9.7)	1.4 (0.8 to 2.5)	801	2.1 (1.0 to 3.2)	3.9 (1.5 to 10.2)	229	0.9 (0.0 to 2.1)	1.8 (0.3 to 11.7)	0.28 (0.15 to 0.51)	0.42 (0.10 to 1.84)
Condom use in past 12 months											
No partners/always	616	1.9 (0.8 to 3.0)	Referent	1055	0.5 (0.0 to 1.0)	Referent	1073	0.4 (0.0 to 0.8)	Referent	0.26 (0.08 to 0.87)	0.82 (0.19 to 3.55)
Sometimes/never	2363	7.8 (6.6 to 9.0)	3.5 (1.9 to 6.5)	3925	2.0 (1.5 to 2.5)	3.4 (1.1 to 10.7)	2970	1.3 (0.7 to 1.8)	2.5 (0.8 to 8.2)	0.26 (0.19 to 0.34)	0.63 (0.38 to 1.03)
Alcohol in past 3 months											
Never/monthly	2570	5.8 (4.8 to 6.8)	Referent	4488	1.5 (1.1 to 1.9)	Referent	3788	1.1 (0.7 to 1.6)	Referent	0.26 (0.19 to 0.35)	0.76 (0.48 to 1.23)
Daily/weekly	398	11.2 (7.8 to 14.6)	1.7 (1.1 to 2.5)	517	2.8 (1.1 to 4.5)	2.1 (1.1 to 4.1)	262	0.4 (0.0 to 1.1)	0.3 (0.0 to 2.2)	0.25 (0.13 to 0.50)	0.14 (0.02 to 1.05)
Prior HIV test											
<1 year	1100	4.6 (3.3 to 5.9)	Referent	1972	1.0 (0.5 to 1.4)	Referent	2379	0.9 (0.4 to 1.3)	Referent	0.21 (0.12 to 0.36)	0.91 (0.45 to 1.82)
1 to 4 years	911	6.3 (4.6 to 7.9)	1.4 (0.9 to 2.0)	2484	1.5 (1.0 to 2.1)	1.6 (0.9 to 3.0)	1174	1.4 (0.5 to 2.2)	1.6 (0.7 to 3.4)	0.24 (0.16 to 0.38)	0.89 (0.44 to 1.83)
≥5 years/no test	895	9.4 (7.3 to 11.4)	2.3 (1.6 to 3.3)	535	5.0 (2.8 to 7.3)	5.9 (3.0 to 11.5)	470	1.5 (0.0 to 3.0)	1.8 (0.6 to 5.3)	0.53 (0.32 to 0.88)	0.29 (0.10 to 0.89)

CI, confidence interval; APR, adjusted prevalence ratio; PR, prevalence ratio.

^a^ Prior diagnostic status of five participants is unknown

^b^ Of all survey participants. Undiagnosed HIV infection = not meeting any of the following conditions: (1) reporting having tested HIV positive previously to standard interview questions, (2) having tested HIV positive at home in a prior round, and (3) having an HIV‐1 RNA concentration <1000 copies/μL. Prevalence of undiagnosed HIV infection was estimated using SURVEYFREQ, weighted to the CHDSS census population by age group, sex, and urban (Chókwè town) or rural (CHDSS villages) residence, and adjusted for within‐household correlation (SAS, version 9.4, SAS Institute)

^c^ All PRs and APRs were weighted to the CHDSS census population by age group, sex, and urban (Chókwè town) or rural (CHDSS villages) residence, and adjusted for within‐household correlation using GENMOD (SAS, version 9.4, SAS Institute). GENMOD APRs also adjusted for age group and sex. All PRs and APRs with a lower bound of the 95% CI ≥1.0 are statistically significant (*p* < 0.05).

In R1 and R3, compared with comparison groups, the prevalence of undiagnosed HIV infection was higher among residents aged 25 to 44 years, and among residents who had sexual partners (R3 only), sometimes or never used condoms, used alcohol at least weekly, and who had never previously tested or last tested ≥5 years ago. In R5, prevalence of undiagnosed HIV infection was similar among all demographic and risk‐behaviour subgroups (Table 3).

## 4 Discussion

In a high‐burden district of Mozambique during 2014 to 2019, annual rounds of HBHTS in 20,000 households newly diagnosed 3711 PLHIV, including nearly 1000 each of men and young adults aged 15 to 24 years, two groups with consistently low diagnostic coverage [1, 2, 3, 4, 5]. After the first two rounds (44,825 home‐based tests; 31,717 facility‐based tests), 59% of residents aged 15 to 59 years had tested at home at least once, the prevalence of undiagnosed HIV infection decreased 75%, and diagnostic coverage among PLHIV increased from 73.8% to 93.0%, exceeding in 2016 the 90% target for 2020 [19]. After another two rounds (32,226 home‐based tests; 46,003 facility‐based tests), demographic and risk‐behaviour disparities in undiagnosed HIV infection were eliminated and diagnostic coverage in CHDSS reached 95% overall, achieving in 2019 the target for 2030 [1]. Findings from CHDSS are consistent with more than 30 studies in sub‐Saharan Africa on high uptake and testing coverage of HBHTS, and with two recent test and treat trials that used two or more rounds of door‐to‐door HBHTS, in addition to standard facility‐based testing, to diagnose >90% of PLHIV in communities in Zambia and South Africa [8, 9, 10, 12, 13].

Comparative effects of HBHTS on diagnostic coverage among PLHIV in Mozambique have not been reported. However, modelled estimates suggest that in Gaza province from 2014 to 2016, diagnostic coverage among residents aged ≥15 years increased an absolute 9% (76% to 85%) among women and 11% (59% to 71%) among men [5, 6]. In contrast, after two rounds of HBHTS in CHDSS (located in Gaza), estimated diagnostic coverage during this period increased an absolute 16% (78% to 94%) among women and 28% (63% to 91%) among men aged 15 to 59 years. During 2014 to 2019, HIV testing strategies in Mozambique were largely restricted to healthcare facilities and for partners and children of PLHIV [15]. Notably, in CHDSS, the prevalence of having last tested for HIV in a healthcare facility did not change (45% in 2014, 40% in 2016, 45% in 2019) as prevalence of having last tested at home increased from 13% (2014) to 45% (2016) and 41% (2019).

Not surprisingly, uptake of HBHTS decreased as the prevalence of having ever and recently tested for HIV increased. Unlike recent trials, HBHTS was conducted programmatically in CHDSS without overarching objectives that all HIV‐negative residents should re‐test annually [20]. Notably, even though the proportion of home tests among young adults increased in every round, after four rounds of HBHTS and standard facility‐based testing, diagnostic coverage among PLHIV aged 15 to 24 years reached only 88%. Additionally, after two rounds of HBHTS, prevalence of undiagnosed infection remained higher in several subgroups including residents with sexual‐ and alcohol‐related risks, and those who had not tested in five or more years. These findings are consistent with recent studies suggesting that to diagnose ≥95% of young adults and eliminate disparities in undiagnosed infection within 1 to 2 rounds of HBHTS, additional community‐based testing strategies are needed such as mobile testing at bars and clubs and use of self‐test kits [7, 9, 10, 20, 21, 22].

During 2014 to 2019 when HIV prevalence in CHDSS was stable at approximately 24%, HBHTS yield of new diagnoses was slightly higher than the prevalence of undiagnosed infection in the first (7.5% vs. 6.6%), third (2.9% vs. 1.7%), and fifth (1.3% vs. 1.1%) rounds. Additionally, HBHTS yield of new diagnoses among residents aged 15 to 24 years in all rounds was at least three‐fold higher among women than men, reflecting underlying HIV gender disparities among young adults in CHDSS and throughout sub‐Saharan Africa [2, 3, 4]. These findings suggest that home‐based testing in CHDSS was an effective population‐based testing strategy as the yield of new HIV diagnoses approximated the underlying prevalence and distribution of undiagnosed infection, even as the prevalence of undiagnosed infection decreased. Although the prevalence of undiagnosed infection in CHDSS decreased 36% (PR 0.64; 95% CI 0.41 to 1.01) between the third and fifth rounds, the reduction was not statistically significant, attributed in part to the diminishing returns of HBHTS. While index and facility‐based testing typically have a higher yield than HBHTS, these strategies alone are insufficient to achieve population‐level testing and diagnostic coverage [7, 8, 9, 10, 22]. Notably, of the four recent trials that have been able to achieve ≥90% diagnostic coverage among PLHIV in sub‐Saharan Africa, all implemented some form of HBHTS [20].

HBHTS achieves high testing coverage in populations by reducing well‐known barriers to testing including costs in transportation, time, and lost work, fear of stigma and discrimination, and unfamiliarity with or negative perceptions about healthcare [7, 8, 9, 10, 20, 23]. Other social and medical benefits of HBHTS include testing of couples and undiagnosed children, normalizing HIV testing and treatment in communities, and diagnosing PLHIV sooner after infection [7, 8, 9, 10, 20]. Nonetheless, despite repeated findings of these benefits and success in achieving high testing and diagnostic coverage, HBHTS is now rarely supported programmatically in sub‐Saharan Africa, primarily due to costs [24].

Early studies suggested costs per person tested were lower for HBHTS (USD $2 to $14) than facility‐based testing (USD $12 to $94) [8, 9, 10, 11]. More recent studies suggest that costs per person tested vary considerably but are generally higher for HBHTS ranging from $6 to $55 USD, than facility‐based testing ranging from $5 to $31 USD [25, 26]. These studies, however, did not consider averted costs. Because HBHTS is superior to facility‐based testing in achieving diagnostic coverage and diagnosing PLHIV earlier, combined with effective linkage‐to‐ART services, HBHTS may be more cost‐effective than facility‐based testing in deaths and disability‐adjusted life years averted [7, 8, 9, 10].

The importance of providing effective linkage services is underscored by over 15 studies in sub‐Saharan Africa, including one in Mozambique, suggesting that only 18%‐51% of PLHIV diagnosed in community settings enrol early in HIV care when referral is the only linkage service [10, 27, 28, 29]. Among all PLHIV aged 15 to 59 years in CHDSS, however, ART coverage increased from 65% in R1 to 88% in R5, and viral load suppression coverage increased from 52% in R1 to 78% in R5 [30]. These findings suggest that CHDSS lay counsellors were effective in linking clients to HIV care and that HBHTS can help make substantial progress towards 95‐95‐95 in high‐burden communities of Mozambique. Similar linkage services have achieved near‐universal linkage to care among community‐diagnosed PLHIV in other sub‐Saharan African countries [10, 23, 27, 28].

The cost‐effectiveness of HBHTS can be improved by incorporating effective measures to control other diseases such as tuberculosis, malaria, and childhood diarrhoea, and increasing both the coverage and impact of high priority HIV prevention programmes such as Determined, Resilient, Empowered, AIDS‐free, Mentored, and Safe (DREAMS) for adolescent and young adult women, and voluntary medical male circumcision (VMMC) for men [31, 32, 33, 34]. To increase DREAMS coverage, HBHTS platforms could be used to identify high‐risk HIV‐negative women for pre‐exposure prophylaxis, and intimate partner violence mitigation and prevention [24, 33]. During the first round of HBHTS alone, over 5000 HIV‐negative women aged 15 to 24 years were identified in CHDSS, many of whom would likely have been eligible for DREAMS [33]. Moreover, after two rounds of HBHTS and linkage services, ART coverage among men increased from 56.5% to 71.6% [30]. Diagnosing and linking HIV‐positive men to ART is the most effective means to prevent HIV infections among young women [24, 33].

The findings in this report are subject to at least five limitations. First, because the evaluation did not include control communities and CHDSS residents who were newly HIV diagnosed in healthcare facilities remains unknown, the effect of HBHTS on diagnostic coverage and prevalence of undiagnosed HIV infection could not be estimated. The effect, however, is expected to be large since in 2014 an estimated 3473 (52,618*0.066) HIV‐positive residents aged 15 to 59 years were undiagnosed, and 2755 (79%) were tested and diagnosed at home in the first two rounds. Second, counts of home‐ and facility‐based tests do not represent unique persons who might test more than once within and across rounds. Also, because patient residence was not recorded on facility test registers, reported facility‐based HIV tests do not represent tests among residents of any geographical area including CHDSS or Chókwè District. Many residents outside CHDSS are known to receive care at Chókwè hospitals and likely contribute to HIV‐test counts. Third, although all prevalence estimates were weighted to the census population, residual bias might reduce the validity of trends among men who were underrepresented in survey rounds 3 and 5. Because of cost considerations, research staff were not able to revisit homes repeatedly in these rounds, and most men did not participate because they were never contacted. Fourth, trends in the prevalence of undiagnosed infection might be biased downwards as proportionally fewer participants in R3 and R5 than R1 reported sexual‐ and alcohol‐related risk behaviours. However, HIV prevalence was similar across rounds, suggesting that this potential bias is likely to be small. Although misclassification might also bias trends in undiagnosed infection, only 12 participants across survey rounds were reclassified as having received a prior HIV diagnosis based on viral load suppression results, suggesting that trends were not affected by misclassification. Finally, CHDSS prevalence of prior HIV diagnosis among PLHIV in 2014 (73.8%) is higher than modelled estimates for Gaza province in 2014 that adjusted for underreporting (69.4%) [5, 6]. Although differences may be attributed to the uncertainty of modelled estimates or bias in our study, higher baseline diagnostic coverage in CHDSS might also be attributed, in part, to HBHTS that was provided at a lower scale in CHDSS before 2014.

## 5 Conclusions

After achieving >90% diagnostic coverage in CHDSS, the prevalence of undiagnosed HIV infection was not statistically significantly reduced with additional rounds of HBHTS. Our findings suggest that sub‐Saharan African countries should consider implementing 1 to 2 rounds of HBHTS in underserved high‐burden communities that have not yet met the UNAIDS 2030 diagnostic coverage target. The United States President’s Emergency Plan for AIDS Relief supports door‐to‐door HBHTS for high‐burden communities with <70% ART coverage [24]. To increase cost‐effectiveness, HIV testing programmes should consider implementing HBHTS with effective linkage services in close collaboration with DREAMS and VMMC prevention programmes, and to the extent possible, with programmes controlling other high‐priority diseases.

## Competing interests

The authors have declared no conflict of interest.

## Authors’ contributions

DM, IC, RN, RT, SP, and SW (alphabetical order) designed and wrote the study protocol and data collection tools. AV, DM, IC, IP, RT, and SW (alphabetical order) acquired funding for the study, and AJ, IC, IM, JB, KH, RT, ST, and VC (alphabetical order) administered the project resources or participant healthcare services. AJ, IC, JB, JC, and RT (alphabetical order) supervised study staff, and DU, IC, and RN (alphabetical order) oversaw data management. RN and SP (alphabetical order) conducted statistical analyses and sample size calculations. DM wrote the first draft of the manuscript, and all authors contributed to and approved the final version of the manuscript for submission.

## Ethical approval

The study protocol was approved by the CHDSS community advisory board, the National AIDS Control Program of the Mozambique Ministry of Health, and the Mozambique National Committee for Bioethics. The study was also reviewed and approved in accordance with CDC human research protection procedures. All residents who tested for HIV and participated in interviews provided written informed consent. Consent forms were administered in Shangaan or Portuguese. HBHTS was provided to all consenting household members including children with parental consent; however, data collection was restricted to residents aged 15 to 59 years in accordance with human subjects’ research approvals.

## Supporting information


**Appendix S1**. Supplementary CHDSS information.

**Table S1**. Home‐based HIV tests and new HIV diagnoses among residents aged 15 to 59 years, by sex and age group, Chókwè Health Demographic Surveillance System, Chókwè District, Mozambique, 2014 to 2019

**Table S2**. Facility‐based HIV tests among patients aged ≥15 years, Chókwè Health Demographic Surveillance System, Chókwè District, Mozambique, 2014 to 2019
