# Supplementary material for: Annual home‐based HIV testing in the Chókwè Health Demographic Surveillance System, Mozambique, 2014 to 2019: serial population‐based survey evaluation
Source: J Int AIDS Soc. 2021 Jul 14;24(7):e25762. doi: 10.1002/jia2.25762 (PMC8278856; doi:10.1002/jia2.25762)
Supplement: Supplementary file 1 — Appendix S1. Supplementary CHDSS information. [file JIA2-24-e25762-s001.docx]

**Chókwè Health Demographic Surveillance System**

**Background**

The Chókwè Health Demographic Surveillance System (CHDSS) includes approximately 100,000 residents of 20,000 households, representing half of the population in Chókwè district, Mozambique. The CHDSS includes all barrios of Chókwè town and seven rural district villages: Barragem, Conhane, Manjangue, Massavasse, Matuba, Muzumuia, and Lionde. Chókwè district is located in Gaza province which has the highest prevalence of HIV infection in the country’s adult population. Chókwè District has the country’s largest irrigation system, and a complex socioeconomic pattern that markedly affects its demographic and health profile. Prone to waterborne infectious diseases, there is an entrenched pattern of male migration to neighboring South Africa in search of employment on which many households economically depend. This migration pattern contributes markedly to the socioeconomic complexity of this district and plays a key role in the district’s epidemiologic profile.

The Chókwè Health Research and Training Center (CITSC), a peripheral research unit of the Mozambique National Institute of Health, established the CHDSS in 2010 with support from the United States President’s Emergency Plan for AIDS Relief and from Family Health International. Since October 2014, CHDSS has been a member of the INDEPTH network. The network includes 45 demographic surveillance centers in 20 countries in Africa, Asia, and the Pacific, covering more than 3.5 million individuals. INDEPTH recommends at least annual visits to all participating households for demographic and vital events surveillance. Although centers (geographic areas) are generally not representative of the populations of their host countries as a whole, they provide access to accurate, high-quality, population-based longitudinal health and demographic data for a well-defined population. Such data can be used to plan and evaluate important demographic and health evaluations.

**Census Objectives and Methods (2010–2020)**

Primary objectives of the CHDSS include monitoring demographic and health indicators in the district’s population through a baseline census, followed by ongoing surveillance of all demographic events, including migration (internal and external), births, deaths, social and economic determinates of health, the burden of HIV and other diseases, and the impact of public health and biomedical services and interventions.

Nearly all (99%) households in the CHDSS participate in annual or biannual rounds of health and demographic census. Each of the approximately 20,000 CHDSS households is geolocated, and each household resident is assigned a permanent identification number. During census rounds, CITSC teams visit all participating households and collect vital events (births, deaths), demographic (sex, age, education, employment), residence, and migratory information on all household members. Additional information is collected on the household itself, including type of construction, type of latrine, and access to electricity and water. CITSC teams also conduct oral autopsies on all reported deaths. Data are entered in real-time into tablets with pre-programmed validation error checking. Electronic census and health-surveillance data are uploaded on a server and shared regularly with the Mozambique Ministry of health and INS.
