# Supplementary material for: Annual home‐based HIV testing in the Chókwè Health Demographic Surveillance System, Mozambique, 2014 to 2019: serial population‐based survey evaluation
Source: J Int AIDS Soc. 2021 Jul 14;24(7):e25762. doi: 10.1002/jia2.25762 (PMC8278856; doi:10.1002/jia2.25762)
Supplement: Supplementary file 2 — Table S1. Home‐based HIV tests and new HIV diagnoses among residents aged 15 to 59 years, by sex and age group, Chókwè Health Demographic Surveillance System, Chókwè District, Mozambique, 2014 to 2019 [file JIA2-24-e25762-s003.docx]

**TABLE S1**. **Home-based HIV tests and new HIV diagnoses among residents aged 15-59 years, by sex and age group, Chókwè Health Demographic Surveillance System, Chókwè District, Mozambique, 2014-2019**

|  | **Round 1**  **(04/2014 – 04/2015)** | | | **Round 2**  **(05/2015 – 01/2016)** | | | **Round 3**  **(03/2016 – 12/2016)** | | | **Round 4**  **(03/2017 – 11/2017)** | | | **Round 5**  **(04/2018 – 03/2019)** | | |
| --- | --- | --- | --- | --- | --- | --- | --- | --- | --- | --- | --- | --- | --- | --- | --- |
|  | **Residents**  **n** | **HIV Tests^a^**  **n (monthly)** | **New Dx^b^**  **n (%)** | **Residents**  **n** | **HIV Tests^a^**  **n (monthly)** | **New Dx^b^**  **n (%)** | **Residents**  **n** | **HIV Tests^a^**  **n (monthly)** | **New Dx^b^**  **n (%)** | **Residents**  **n** | **HIV Tests^a^**  **n (monthly)** | **New Dx^b^**  **n (%)** | **Residents**  **n** | **HIV Tests^a^**  **n (monthly)** | **New Dx^b^**  **n (%)** |
| Total | 52618 | 24979 (1921) | 1879 (7.5) | 49032 | 19846 (2205) | 876 (4.4) | 46965 | 17880 (1788) | 524 (2.9) | 44912 | 14346 (1594) | 231 (1.6) | 46923 | 15461 (1288) | 201 (1.3) |
| Sex |  |  |  |  |  |  |  |  |  |  |  |  |  |  |  |
| Women | 32815 | 17190 (1322) | 1422 (8.3) | 30580 | 13344 (1483) | 600 (4.5) | 29021 | 12000 (1200) | 394 (3.3) | 27570 | 9499 (1055) | 158 (1.7) | 28796 | 10407 (867) | 157 (1.5) |
| Men | 19803 | 7789 (599) | 457 (5.9) | 18452 | 6502 (722) | 276 (4.2) | 17944 | 5880 (588) | 130 (2.2) | 17342 | 4847 (539) | 73 (1.5) | 18127 | 5054 (421) | 44 (0.9) |
| Age group |  |  |  |  |  |  |  |  |  |  |  |  |  |  |  |
| 15-24 | 22514 | 9760 (751) | 423 (4.3) | 20338 | 8984 (998) | 230 (2.6) | 19706 | 8489 (849) | 152 (1.8) | 18648 | 7396 (822) | 77 (1.0) | 20222 | 8585 (715) | 82 (1.0) |
| 25-44 | 21859 | 10504 (808) | 1111 (10.6) | 20708 | 7344 (816) | 522 (7.1) | 19530 | 6431 (643) | 304 (4.7) | 18723 | 4767 (530) | 117 (2.5) | 19017 | 4836 (403) | 88 (1.8) |
| 45-59 | 8245 | 4715 (363) | 345 (7.3) | 7986 | 3518 (391) | 124 (3.5) | 7729 | 2960 (296) | 68 (2.3) | 7541 | 2183 (243) | 37 (1.7) | 7684 | 2040 (170) | 31 (1.5) |
| Women |  |  |  |  |  |  |  |  |  |  |  |  |  |  |  |
| 15-24 | 13154 | 5912 (455) | 362 (6.1) | 11774 | 5295 (588) | 187 (3.5) | 11117 | 4969 (497) | 131 (2.6) | 10266 | 4174 (464) | 60 (1.4) | 11240 | 5110 (426) | 74 (1.4) |
| 25-44 | 14174 | 7813 (601) | 795 (10.2) | 13440 | 5452 (606) | 325 (6.0) | 12688 | 4779 (478) | 208 (4.4) | 12150 | 3623 (403) | 75 (2.1) | 12308 | 3706 (309) | 67 (1.8) |
| 45-59 | 5487 | 3465 (267) | 265 (7.6) | 5366 | 2597 (289) | 88 (3.4) | 5216 | 2252 (225) | 55 (2.4) | 5154 | 1702 (189) | 23 (1.4) | 5248 | 1591 (133) | 16 (1.0) |
| Men |  |  |  |  |  |  |  |  |  |  |  |  |  |  |  |
| 15-24 | 9360 | 3848 (296) | 61 (1.6) | 8564 | 3689 (410) | 43 (1.2) | 8589 | 3520 (352) | 21 (0.6) | 8382 | 3222 (358) | 17 (0.5) | 8982 | 3475 (290) | 8 (0.2) |
| 25-44 | 7685 | 2691 (207) | 316 (11.7) | 7268 | 1892 (210) | 197 (10.4) | 6842 | 1652 (165) | 96 (5.8) | 6573 | 1144 (127) | 42 (3.7) | 6709 | 1130 (94) | 21 (1.9) |
| 45-59 | 2758 | 1250 (96) | 80 (6.4) | 2620 | 921 (102) | 36 (3.9) | 2513 | 708 (71) | 13 (1.8) | 2387 | 481 (53) | 14 (2.9) | 2436 | 449 (37) | 15 (3.3) |
| Residence |  |  |  |  |  |  |  |  |  |  |  |  |  |  |  |
| Chókwè town | 33766 | 15612 (1201) | 1050 (6.7) | 31564 | 11865 (1318) | 550 (4.6) | 30012 | 10844 (1084) | 325 (3.0) | 28778 | 8715 (968) | 152 (1.7) | 30077 | 9441 (787) | 134 (1.4) |
| Dist. villages | 18852 | 9330 (718) | 829 (8.9) | 17468 | 7903 (878) | 319 (4.0) | 16953 | 7025 (703) | 199 (2.8) | 16134 | 5624 (625) | 78 (1.4) | 16846 | 6002 (500) | 66 (1.1) |

New Dx, New HIV diagnosis; Dist., District.

^a^Each round, lay counselors certified to provide rapid HIV testing and counseling visited approximately 20,000 homes that compose the CHDSS and offered home-based HIV testing services to encountered household members.

^b^New HIV diagnosis = not meeting any of the following conditions: (1) reporting having tested HIV-positive previously to standard interview questions, (2) having tested HIV-positive at home in a prior round, and (3) having an HIV-1 RNA concentration <1,000 copies/μL.
