# Supplementary material for: Annual home‐based HIV testing in the Chókwè Health Demographic Surveillance System, Mozambique, 2014 to 2019: serial population‐based survey evaluation
Source: J Int AIDS Soc. 2021 Jul 14;24(7):e25762. doi: 10.1002/jia2.25762 (PMC8278856; doi:10.1002/jia2.25762)
Supplement: Supplementary file 3 — Table S2. Facility‐based HIV tests among patients aged ≥15 years, Chókwè Health Demographic Surveillance System, Chókwè District, Mozambique, 2014 to 2019 [file JIA2-24-e25762-s002.docx]

**TABLE S2**. **Facility-based HIV tests among patients aged >15 years, Chókwè Health Demographic Surveillance System,** **Chókwè District, Mozambique, 2014-2019.^a^**

|  | **HIV Tests**  **(04/2014 – 03/2015)**  **n** | **HIV Tests**  **(04/2015 – 03/2016)**  **n** | **HIV Tests**  **(04/2016 – 03/2017)**  **n** | **HIV Tests**  **(04/2017 – 03/2018)**  **n** | **HIV Tests**  **(04/2018 – 03/2019)**  **n** |
| --- | --- | --- | --- | --- | --- |
| **Total** | 9778 | 21939 | 19457 | 26546 | 35607 |
| Chókwè Town |  |  |  |  |  |
| 6 Bairro Health Post | 0 | 0 | 195 | 502 | 1506 |
| Carmelo Hospital | 2585 | 2753 | 1611 | 1090 | 2129 |
| Chókwè Health Center | 855 | 2149 | 1512 | 2097 | 3739 |
| Chókwè Rural Hospital | 3986 | 11396 | 8271 | 12695 | 14561 |
| Villages |  |  |  |  |  |
| Barragem Health Center | 0 | 856 | 2954 | 1503 | 2478 |
| Conhane Health Center | 57 | 658 | 874 | 1458 | 2286 |
| Lionde Health Center | 1074 | 2071 | 1882 | 2622 | 3647 |
| Manjangue Health Center | 1001 | 1174 | 1333 | 3016 | 3529 |
| Massavasse Health Post | 220 | 882 | 825 | 1563 | 1732 |

^a^Obtained from routine Ministry of Health reports. HIV tests do not represent unique patients who can retest within the same or different healthcare facilities. Because patient residence was not recorded on test registers, HIV tests also do not represent tests among residents of any geographic area including the Chókwè Health Demographic Surveillance System or Chókwè District. New HIV diagnoses among CHDSS residents tested at healthcare facilities was not measured and is not reportable.
